# Effect of Cabergoline on weight and glucose metabolism in patients with acromegaly

**DOI:** 10.1007/s40618-024-02396-1

**Published:** 2024-05-24

**Authors:** E. Varaldo, N. Prencipe, C. Bona, D. Cuboni, L. S. Aversa, M. Sibilla, F. Bioletto, A. M. Berton, C. Gramaglia, V. Gasco, E. Ghigo, S. Grottoli

**Affiliations:** 1 Division of Endocrinology, Diabetology and Metabolism, Department of Medical Sciences, University of Turin, Corso Dogliotti, 14, 10126 Turin, Italy; 2 Division of Endocrinology, Diabetology and Metabolism, S. Croce and Carle Cuneo Hospital, Cuneo, Italy

**Keywords:** Cabergoline, Dopamine agonist, Acromegaly, Weight loss, Diabetes mellitus, Impaired glucose metabolism

## Abstract

**Purpose:**

Cabergoline (CAB) has shown to have benefic effects on the metabolism in different clinical settings but its metabolic role in acromegaly disease has not been studied yet. Aim of our study was to evaluate the impact of CAB on glucose metabolism and weight in patients with acromegaly.

**Methods:**

All patients with acromegaly undergoing continuous treatment with CAB for at least 6 months were retrospectively screened. Exclusion criteria were discontinuation of CAB for more than one month, change of antidiabetic or other therapy for acromegaly, concomitant untreated hormonal deficiency, initiation of pregnancy and/or breastfeeding. All patients were evaluated in terms of biochemical disease control, glucose metabolism and weight at baseline (T0) and after the introduction of CAB therapy at 6 (T6) and 12 months (T12).

**Results:**

Twenty-six patients (15 females and 11 males) were evaluated at T0 and T6 and 19 patients (12 females and 7 males) were also evaluated at T12. Insulin-like growth factor I (IGF-I) and prolactin (PRL) levels were significantly lower at T6 and T12 compared to baseline (p < 0.001 for IGF-I, p < 0.05 for PRL) even if no further differences were observed between T12 and T6. Considering the entire cohort, no differences were appreciated regarding the metabolic parameters but a significant reduction in weight and body mass index (BMI) was observed at both T6 (p = 0.009 for weight, p = 0.021 for BMI) and T12 (p = 0.014 for weight, p = 0.017 for BMI) compared to baseline.

**Conclusion:**

Our results confirm the efficacy of CAB in providing a significant improvement in the biochemical disease control but do not demonstrate a marked benefit on glucose metabolism of acromegaly patients. In such patients, CAB appears to have a rapid effect on weight and BMI, with significant changes noticeable as early as 6 months and persisting for at least 12 months.

## Introduction

Acromegaly is a rare condition characterized by an overproduction of growth hormone (GH) and elevated levels of insulin-like growth factor I (IGF-I), typically secondary to a pituitary adenoma.

The diagnosis is established by IGF-I levels > 1.3 times the upper limit of normal (ULN) and GH nadir levels > 1 μg/L, or 0.4 μg/L in case of an ultrasensitive method, during oral glucose tolerance test (OGTT) [1, 2].

The first-line treatment is neurosurgery, but several medical therapies are available, including somatostatin analogs (SSAs), GH receptor antagonist (pegvisomant, PEG), and dopamine agonists (DAs).

Cabergoline (CAB) is a DA that has long been used off-label for the treatment of acromegaly due to its proven efficacy in normalizing IGF-I levels in one-third of cases in monotherapy and in up to 40–50% when used in combination with SSAs or PEG [3–5].

In acromegaly patients, the presence of metabolic complications is far from being uncommon: at diagnosis, 20–56% of patients have confirmed diabetes mellitus (DM), while 16–46% present impaired glucose metabolism (IGM) [6]. Adequate management of these comorbidities is crucial as they significantly affect the survival of these individuals [7] and as a consequence, the metabolic impact of the different therapeutic options available is of particular interest.

DAs have shown in various clinical settings to have a positive impact on the metabolic profile of patients and in 2009, the Food and Drug Administration (FDA) approved the use of bromocriptine as an antidiabetic drug due to its significant modulation at the pancreatic level, able to regulate insulin secretion and to preserve β-cell functionality [8].

Although CAB is still off-label for treating DM, encouraging outcomes have also been demonstrated for this molecule in patients with persistent fasting hyperglycemia [9].

The dopaminergic tone is involved in the complex processes that regulate the sense of appetite, and it has been shown that in overweight and obese patients, the reduced expression of the D2-receptor with consequent deficit in dopaminergic signaling, may play a role in compulsive behaviors leading to food seeking and ultimately weight gain [8].

Given that this scenario is common in cases of hyperprolactinemia (hyperPRL), several researchers have studied the effect that DAs have on the metabolism of these patients.

It has been shown that CAB can significantly reduce fasting insulin levels and HOMA-IR (homeostatic model assessment of insulin resistance) in normal-weight, overweight, and obese patients even before any clinical evidence of weight loss, suggesting that the improvement in the metabolic profile is partly independent of weight loss and more likely a consequence of its central dopaminergic action [10].

Similar results have been demonstrated in obese subjects without hyperPRL, where treatment with CAB stabilized HOMA-IR and showed improvement in terms of blood glucose levels after OGTT, regardless of weight changes [11].

However, it is in the treatment of prolactinomas that the metabolic effects of CAB can be better appreciated [12]. Hyperprolactinemic patients show significant metabolic alterations, which are associated with a higher incidence of obesity and metabolic syndrome compared to the general population of the same age [13].

The treatment with CAB allows a significant improvement in the metabolic profile, particularly through a marked reduction in HbA1c and HOMA-IR already after 6 months, regardless of possible weight changes, with even more significant results if CAB is used at high doses and for a prolonged period (> 12 months) [10].

Moreover, the comparison between patients treated with CAB and those undergone neurosurgery showed that the improvement in metabolic parameters is also independent of prolactin (PRL) [14]. In this regard, CAB appears to offer an intrinsic benefit on the glycolipid metabolism of these patients.

Lastly, the benefic effect of DA on the glycemic profile is marked even in patients with Cushing's disease, regardless of the underlying disease control [15].

Given these pieces of evidence, it can be hypothesized that in acromegaly patients, CAB could be a significant modulator of insulin sensitivity through the same mechanisms. Considering the high prevalence of DM and IGM in acromegaly subjects and their impact on prognosis and given the established role of CAB therapy in acromegaly, it seems appropriate to evaluate its metabolic effects in this population.

All this considered, aim of our study was to evaluate the effects of CAB therapy on disease control, glucose metabolism, and any weight changes in acromegaly patients, both as monotherapy and in combination with SSAs or PEG.

## Materials and methods

This retrospective monocentric study was conducted at the Division of Endocrinology, Diabetology and Metabolism of the University Hospital "Città della Salute e della Scienza di Torino " (Turin, Italy) and all patients diagnosed with acromegaly and treated with CAB were screened.

Inclusion criteria were continuous treatment with CAB for at least 6 months and availability of glucose metabolism assessments according to the study protocol. Exclusion criteria were discontinuation of CAB for more than one month, change of antidiabetic or other therapy for acromegaly, untreated hormonal deficiency, initiation of pregnancy and/or breastfeeding.

All patients were evaluated in terms of biochemical disease control (IGF-I, GH and PRL levels), glucose metabolism (fasting blood glucose [FBG] and HbA1c) and weight at baseline (T0) and after the introduction of CAB therapy at 6 (T6) and 12 months (T12).

Demographic and anamnestic details concerning the characteristics of pituitary adenomas (including potential co-secretion), anthropometric measurements (weight, height, and body mass index [BMI]), concomitant hormonal deficiencies and disease-related information were retrospectively collected through the analysis of medical records.

None of the women enrolled in the study were on either hormonal replacement therapy or combined oral contraceptive.

The diagnosis of impaired fasting glucose (IFG), impaired glucose tolerance (IGT) and DM was made according to international guidelines based on HbA1c values, FBG, and OGTT [16].

According to World Health Organization (WHO) classification, normal weight was defined by a BMI between 18.5 and 24.9 kg/m^2^, overweight was defined by a BMI between 25 and 29.9 kg/m^2^ and obesity was defined by a BMI ≥ 30 kg/m^2^.

The study was approved by the Local Ethics Committee and was in accordance with the principles of the Declaration of Helsinki. Written informed consent was obtained from all study participants.

### Laboratory measurements

All blood samples were obtained between 08:00 and 11:00 am after a minimum 12-h fasting period.

At T0, serum GH was determined as the mean value during a 3-h profile (blood samples collected every 30 min for a total of 7 points). Serum PRL was assessed using a 2-point profile (first sample at + 30’ and second at + 60’), excluding any influence from venipuncture stress [17]: in particular, an intravenous cannula was inserted into an antecubital vein and maintained patent by slow infusion of 0.9% normal saline solution. In all subjects polyethylene glycol precipitation for macroprolactinemia (macroPRL) was assayed on basal sample, and the lowest PRL value was considered for the analysis. Patients remained recumbent throughout the procedure.

At T6 and T12, PRL and GH levels, along with other biochemical evaluations, were measured in a single determination. GH levels were not assayed in subjects undergoing PEG therapy.

Serum GH levels (μg/L) were measured in duplicate by IRMA method (IRMA GH, Beckman Coulter, Czech Republic). The IRMA assay of GH is a sandwich-type assay. The calibrators are calibrated against the international standard WHO 2nd IS 98/574 in human serum. The sensitivity of the assay was 0.033 µg/L; intra- and inter-assay coefficients of variation (CV) were 2.4–6.5% and 9.0–14.0%, respectively.

Serum IGF-I levels (μg/L) were measured in duplicate by RIA method (SM-C-RIA-CT, DIAsource Immuno Assays, Belgium) after acid–ethanol extraction to avoid interference by binding proteins. The sensitivity of the method was 0.25 μg/L; intra- and inter-assay CV were 4.5–7.0% and 6.8–14.9%, respectively.

Considering the variability in normal values based on patients’ age, IGF-I levels were additionally standardized according to ULN.

PRL assay was determined by a sandwich immunoassay, using COBASe601 (Roche Diagnostic S.p.a. Monza, Italy) automated method. Intra- and inter-assay CV were, respectively, 2–4.7% for low control and 1.7–4.4% for high control. Normal values in men were 3.5–15.2 µg/L and in women 5–26 µg/L. MacroPRL was defined as positive in case of a recovery rate < 40% of PRL after polyethylene glycol precipitation. Suppression of PRL levels was defined as the presence of PRL levels below 5 µg/L.

Finally, FBG and HbA1c were assayed in plasma according to the automated methods currently used in the analysis laboratory of our center.

### Statistical analysis

Normally and non-normally distributed variables were expressed as mean and standard deviation (SD) or median and interquartile range (IQR), respectively, while categorical data were expressed as counts and percent. Normality was assessed using the Shapiro–Wilk test.

The analysis of longitudinal differences over time were performed with Student's t test for paired or independent samples in the case of variables with normal distribution; to highlight the differences between the median values of non-normally distributed variables Wilcoxon and Mann–Whitney tests were used when appropriate.

A cut-off of p value < 0.05 was considered as statistically significant. Statistical analysis was performed using MedCalc® (Statistical Software version 20.007, MedCalc Software Ltd, Ostend, Belgium). Figures were made using GraphPad Prism (version 8.0.2; GraphPad Software Inc., La Jolla, California).

## Results

### Patient characteristics at baseline (T0)

Thirty-nine patients undergoing CAB treatment for a continuous period of at least 6 months were initially screened. Subsequently, 11 subjects were excluded from the sample as they either changed their antidiabetic therapy or discontinued CAB treatment for over a month during the study, and 2 more patients were excluded because they were affected by untreated hypogonadotropic hypogonadism.

**Fig. 1:**
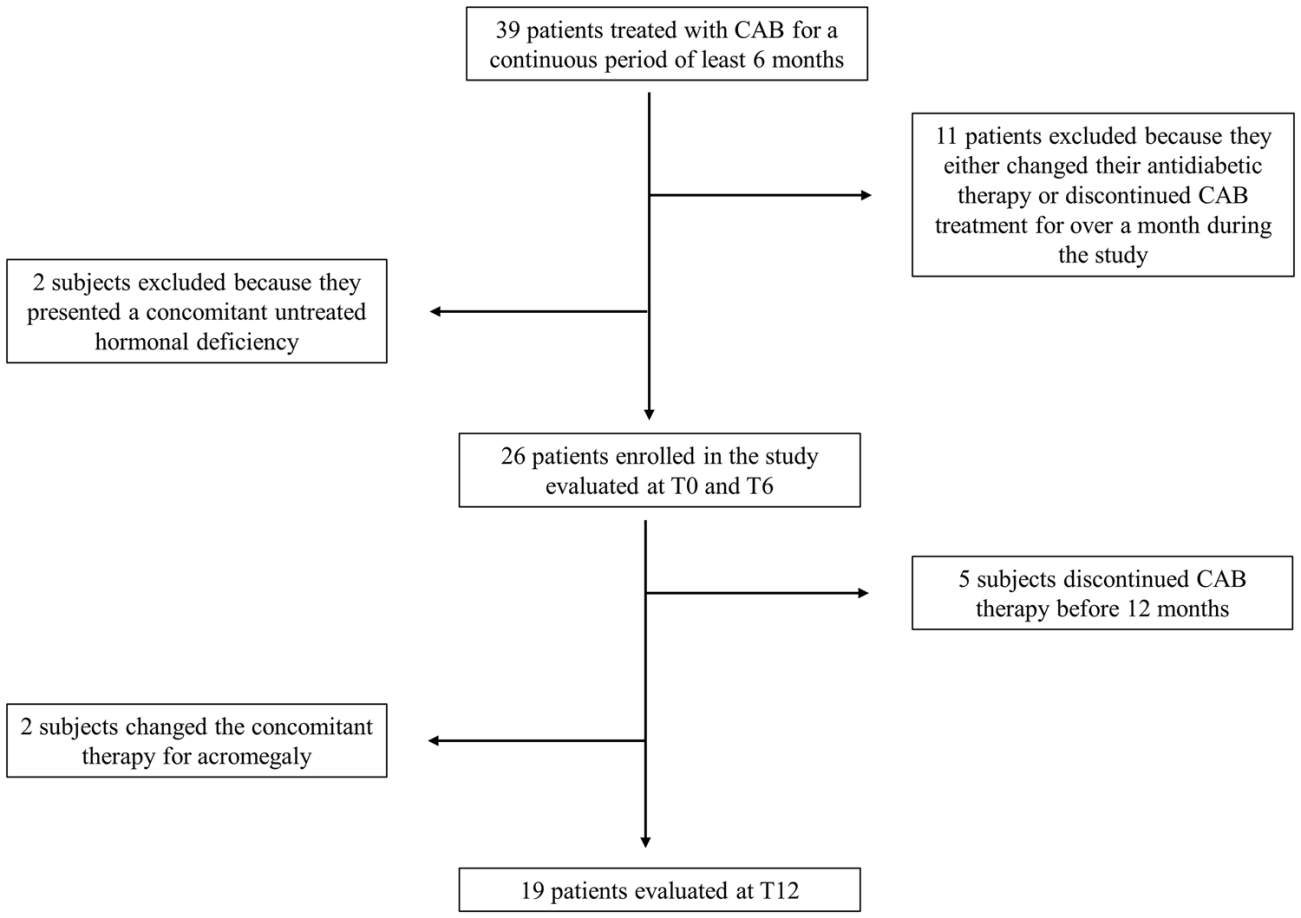
Enrollment process flow-chart. *CAB* Cabergoline, *T0* Baseline, *T6* 6-months evaluation, *T12* 12-months evaluation

Hence, a total of 26 patients diagnosed with acromegaly (15 females [58%] and 11 males [42%]) were examined (Fig. 1) with a median age at diagnosis of 54.5 (42–60) years and at enrollment of 58.5 (42–64) years.

Among these individuals, 15 patients (53.8%) had previously undergone neurosurgery (median latency 25 [17–49] months) and 2 (7.7%) had received adjuvant radiotherapy (8 months and 125 months before, respectively). Among patients undergone neurosurgery, 7 had a GH-secreting adenoma and 6 had a GH/PRL-secreting adenoma. In two patients, it was not possible to define the histological subtype because the collected material was insufficient.

Two subjects had central hypothyroidism, one had central adrenal insufficiency, and three male patients presented central hypogonadism: all patients had been on adequate replacement therapy at a stable dose for at least 4 months with levothyroxine, at least 6 months with testosterone and more than 24 months with cortisone acetate. Regarding the women, 12 out of 15 (80%) were in menopause, while the remaining 3 had regular menstrual cycles.

Thus, 9 patients (34.6%) were on CAB monotherapy, 14 (53.9%) were undergoing adjunctive therapy with SSA, and 3 (11.5%) were receiving a combination with PEG. Ongoing medical treatment was administered at a stable dose for at least 6 months.

CAB was administered orally, starting with a weekly dose of 0.5 mg, and dose adjustment was made based on serum IGF-I levels at follow-up at 3, 6 or 12 months. The maximum dosage achieved was 3.5 mg weekly in one subject.

SSA was administered intramuscularly every 28 days (17 patients were treated with octreotide, at a minimum dosage of 10 mg/28 days and a maximum of 30 mg/28 days; 2 patients were treated with lanreotide at a minimum dosage of 90 mg/28 days and a maximum of 120 mg/28 days).

PEG was administered by subcutaneous injection (3 subjects, dosage range 5–30 mg/day).

The clinical, metabolic and hormonal parameters in the patient cohort at T0, T6 and T12 are shown in Table 1 and 2.

**Table 1:** Clinical, metabolic and hormonal parameters in obese, overweight, normal-weight patients and in the whole cohort at baseline (T0), 6 (T6) and 12 (T12) months evaluation. Data are expressed as mean ± standard deviation (SD) or median and interquartile range (IQR) or n (%)

	Baseline (T0)	6 months (T6)	T6 vs T0 (p)	12 months (T12)	T12 vs T6 (p)	T12 vs T0 (p)
Whole cohort	n = 26	n = 26		n = 19		
IGF-I, *μg/L*	419.0 (309.0–541.0)	285.0 (225.0–458.0)	** < 0.001**	285.0 (168.3–311.0)	0.070	** < 0.001**
IGF-I / ULN	1.41 (1.11–1.97)	1.06 (0.83–1.22)	** < 0.001**	0.95 (0.66–1.03)	0.077	** < 0.001**
GH, *μg/L*	2.4 (0.8–3.8)	1.3 (0.7–3.5)	0.073	1.0 (0.7–2.8)	0.460	0.301
PRL, *μg/L*	5.5 (2.4–12.9)	0.6 (0.2–2.0)	** < 0.001**	0.9 (0.2–2.9)	0.499	**0.039**
Patients undergone neurosurgery,* n (%)*	15	15	–	11	–	–
Hystological examination (among patients undergone neurosurgery), *n (%)*						
• GH	7﻿ (46.7)	7 (46.7)	–	6 (54.5)	–	–
• GH/PRL	6 (40)	6 (40)		3 (27.3)		
• Non-diagnostic (insufficient material)	2 (13.3)	2		2 (182)		
Cabergoline monotherapy,* n (%)*	9 (34.6)	9 (34.6)	–	6 (31.6)	-	–
Cabergoline + SSA, *n (%)*	14 (53.8)	14 (53.8)	–	11 (57.9)	–	–
Cabergoline + PEG, *n (%)*	3 (11.6)	3 (11.6)	–	2 (10.5)	–	–
Cabergoline, *mg* *median dose (range)*	–	1 (0.25–3.5)	–	1 (0.25–3.5)	0.208	–
Glucose, *mg/dL*	96 (89–108)	95 (81–109)	0.820	91 (78–102)	0.249	0.112
HbA1c, *%*	5.9 (5.7–6.4)	5.9 (5.5–6.3)	0.962	6.0 (5.6–6.4)	0.469	0.660
Weight, *kg*	76.9 ± 14.8	75.1 ± 15.5	**0.009**	76.1 ± 15.8	0.929	**0.014**
BMI, *kg/m*^*2*^	27.1 ± 3.7	26.5 ± 3.7	**0.021**	26.9 ± 4.0	0.791	**0.017**
DM, *n (%)*	6 (23.1)	6 (23.1)	–	5 (26.3)	–	**-**
IGM, *n (%)*	14 (53.8)	14 (53.8)	–	9 (47.4)	–	–
Obese patients	n = 5	n = 5		n = 5		
Glucose, *mg/dL*	89 (82–110)	87 (81–109)	0.686	80 (76–104)	0.281	0.500
HbA1c, *%*	5.9 (5.5–6.1)	5.7 (5.5–6.3)	0.782	5.6 (5.3–6.4)	0.686	0.787
Weight, *kg*	93.8 ± 12.5	91.8 ± 14.0	0.075	93 ± 14.4	0.324	0.660
BMI, *kg/m*^*2*^	33.0 ± 1.8	32.3 ± 1.3	0.107	32.6 ± 1.3	0.460	0.601
DM, *n (%)*	1 (20)	1 (20)	–	1 (20)	–	–
IGM, *n (%)*	2 (40)	2 (40)	–	2 (40)	–	–
Overweight patients	n = 13	n = 10		n = 7		
Glucose, *mg/dL*	99 (92–117)	101 (81–117)	0.754	97 (88–102)	0.285	0.139
HbA1c, *%*	6.0 (5.7–6.5)	6.1 (5.9–6.5)	0.636	6.3 (5.6–6.4)	0.529	0.779
Weight, *kg*	77.0 ± 10.6	74.6 ± 12.8	**0.041**	71.9 ± 12.4	0.671	**0.032**
BMI, *kg/m*^*2*^	26.7 ± 1.3	25.7 ± 1.8	0.059	25.5 ± 1.8	0.738	**0.043**
DM, *n (%)*	4 (30.8)	2 (20)	–	2 (28.6)	–	–
IGM, *n (%)*	7 (53.8)	6 (60)	–	3 (71.4)	–	–
Normal-weight patients	n = 8	n = 11		n = 7		
Glucose, *mg/dL*	93 (85–101)	88 (81–105)	0.917	92 (86–96)	0.500	0.345
HbA1c, *%*	5.8 (5.7–6.4)	5.7 (5.4–6.0)	0.225	6.0 (6.0–6.0)	0.493	0.465
Weight, *kg*	62.7 ± 9.4	62.4 ± 8.9	0.635	65.3 ± 8.7	0.391	0.235
BMI, *kg/m*^*2*^	23.1 ± 1.5	23.1 ± 1.5	0.943	23.2 ± 1.7	0.423	0.220
DM, *n (%)*	1 (12.5)	3 (27.3)	–	2 (42.9)	–	–
IGM, *n (%)*	5 (62.5)	6 (54.5)	–	4 (57.1)	–	–

The numbers in bold indicate significant values (p < 0.05)

*IGF-I* Insulin-like growth factor I, *ULN* Upper limit of normality, *GH* Growth hormone, *PRL* Prolactin, *BMI* Body mass index, *DM* Diabetes mellitus, *IGM* Impaired glucose metabolism, *SSA* Somatostatin analog, *PEG* Pegvisomant

**Table 2:** Metabolic parameters in patients with diabetes mellitus, impaired glucose metabolism (IGM) and normal glycemic status at baseline (T0), 6 (T6) and 12 (T12) months evaluation. Data are expressed as mean ± standard deviation (SD) or median and interquartile range (IQR)

	Baseline (T0)	6 months (T6)	T6 vs T0 (p)	12 months (T12)	T12 vs T6 (p)	T12 vs T0 (p)
Diabetic	n = 6	n = 6		n = 5		
Glucose, *mg/dL*	114 (91–142)	129 (81–142)	0.600	116 (95–130)	0.500	0.500
HbA1c, *%*	6.6 (6.4–6.8)	6.8 (6.1–7.2)	0.345	6.6 (6.2–7.7)	0.106	0.273
Weight, *kg*	71.0 ± 10.2	68.2 ± 11.8	0.137	67.4 ± 10.3	0.546	**0.042**
BMI, *kg/m*^*2*^	27.0 ± 4.8	25.7 ± 4.5	0.136	26.3 ± 4.6	0.534	0.056
IGM	n = 14	n = 14		n = 9		
Glucose, *mg/dL*	101 (94–108)	98 (86–107)	0.929	93 (89–102)	0.441	0.107
HbA1c, *%*	5.9 (5.8–6.3)	5.9 (5.8–6.2)	0.441	6.1 (5.8–6.3)	0.612	0.151
Weight, *kg*	75.8 ± 12.4	73.5 ± 12.7	**0.018**	71.7 ± 12.6	0.109	**0.020**
BMI, *kg/m*^*2*^	26.9 ± 3.1	26.0 ± 3.0	**0.037**	26.0 ± 3.5	0.109	**0.033**
Normal glycemia	n = 6	n = 6		n = 5		
Glucose, *mg/dL*	88 (83–92)	84 (79–88)	0.094	77 (75–81)	**0.040**	**0.043**
HbA1c, *%*	5.5 (5.3–5.5)	5.5 (5.3–5.5)	0.958	5.3 (5.1–5.7)	0.935	0.500
Weight, *kg*	85.0 ± 21.1	85.4 ± 20.5	0.456	84.3 ± 15.0	0.495	0.495
BMI, *kg/m*^*2*^	27.5 ± 4.4	27.7 ± 4.1	0.415	29.1 ± 4.0	0.548	0.510

The numbers in bold indicate significant values (p < 0.05)

*BMI* Body mass index

Most of the enrolled subjects were either overweight (13 patients, 50%) or obese (5 patients, 19.2%), while normal-weight patients were 8 (30.8%).

Six patients (23.1%) had DM, 14 patients exhibited IGM (8 with IFG [30.7%] and 6 with IGT [23.1%]) while 6 patients (23.1%) were euglycemic. Three subjects with DM were on nutrition therapy and 3 patients were taking oral anti-diabetic drugs (2 subjects on metformin monotherapy and one subject on metformin and dipeptidyl peptidase IV inhibitor therapy) at a stable dose for at least 6 months and no one was treated with either insulin or glucagon-like peptide 1 receptor agonist (GLP1-RA) or sodium-glucose cotransporter-2 inhibitor (SGLT2-i).

At T0, all patients exhibited IGF-I levels elevated for their age (419.0 [309.0–541.0] μg/L corresponding to 1.41 [1.11–1.97] xULN), and 16/23 (69.6%) had GH levels > 1 μg/L (median 2.4 [0.8–3.8] μg/L). Twenty patients had either normal or suppressed PRL levels and 3 had elevated levels (median PRL 5.5 [2.4–12.9] μg/L; range 0.1–717 μg/L) while PRL values were unavailable for 3 subjects.

Finally, no differences were observed in terms of IGF-I, GH, and PRL levels among the three groups considering glycemic status.

### 6-month evaluation

At T6, at a median dose of CAB of 1 (1–2; range 0.25–3.5) mg/week, both IGF-I (285.0 [225.0–458.0] μg/L, p < 0.001) and PRL (0.6 [0.2–2.0] μg/L, p < 0.001) levels were significantly reduced compared to T0 while no significant differences were observed in GH levels (1.3 [0.7–3.5] μg/L, p = 0.073).

Twelve patients (46.2%) had normalized IGF-I values, and all subjects had either normalized (n = 3) or suppressed (n = 23) PRL levels (range 0.1–8.6 μg/L).

No differences were appreciated regarding the metabolic parameters but a significant reduction in both weight (p = 0.009) and BMI was observed (p = 0.021): as a result, the number of normal-weight individuals increased to 11, those overweight decreased to 10, while obese subjects remained 5. However, no differences were observed in terms of percentage change in weight among the 3 groups.

Considering the degree of glycemic alteration at baseline, a notable decrease in body weight was noticed solely in patients with IGM (Table 2). However, no significant differences were observed between the three groups in the rate of weight loss, nor in the change in FBG and HbA1c levels.

Taking into account the adenomatous subtype, weight loss was significant in patients with a GH-secreting adenoma (p = 0.049) while the same result was not observed in patients with a GH/PRL adenoma (p = 0.126); no differences were observed between the 2 groups in terms of disease control.

Stratifying based on the ongoing therapy a significant weight (T0 vs T6, 78.4 ± 12.7 vs 75.9 ± 13.3 kg, p = 0.006) and BMI (T0 vs T6, 28.3 ± 3.9 vs 27.4 ± 3.9 kg/m^2^, p = 0.011) reduction was appreciated only in the subgroup undergoing combination therapy with SSA. No differences were observed in relation to FBG and HbA1c.

Patients who had inhibited PRL levels at 6 months from the start of CAB therapy showed a significant weight (p = 0.008) and BMI (p = 0.021) reduction but did not exhibit significant differences in glycemic profile. Likewise, patients with normalized IGF-I levels did not show a significant weight and BMI reduction or improvement in glycemic control compared to patients with active disease.

Finally, significant weight and BMI reduction were observed in both males and females without any difference between the two sexes. Similarly, no significant difference in the reduction of BG and/or HbA1c was observed between the two sexes.

On the other hand, age did not significantly impact glycemic or weight variation either.

### 12-month evaluation

Of the initial sample population, 5 subjects discontinued CAB therapy before 12 months, while an additional 2 subjects were later excluded from subsequent analysis because they changed the concomitant therapy for acromegaly. This resulted in 19 patients (7 [37%] males and 12 [63%] females) evaluated at T12.

At T12, at a median dose of CAB of 1 (1–2; range 0.25–3.5) mg/week, IGF-I (285.0 [168.3–311.0] μg/L, p < 0.001) and PRL (0.9 [0.2–2.9]) μg/L, p = 0.039) levels were significantly reduced compared to T0 while no differences were observed compared to T6. Moreover, no differences were observed in GH levels compared to both T0 and T6.

Fourteen out of 19 patients (73.7%) had normalized IGF-I levels, and 16 out of 19 patients (84.2%) had suppressed PRL levels.

In the entire cohort, no significant differences were demonstrated in terms of FBG and HbA1c compared to either T0 or T6. However, when dividing subjects into diabetic and non-diabetic groups, a significant reduction in FBG compared to T0 was observed only in the non-diabetic subgroup (T0 vs T12, 93 [88–103] vs 88 [77–96] mg/dL, p = 0.013), specifically in patients with a normal glycemic status, while no significant variations were observed in the other analyzed variables.

**Fig. 2:**
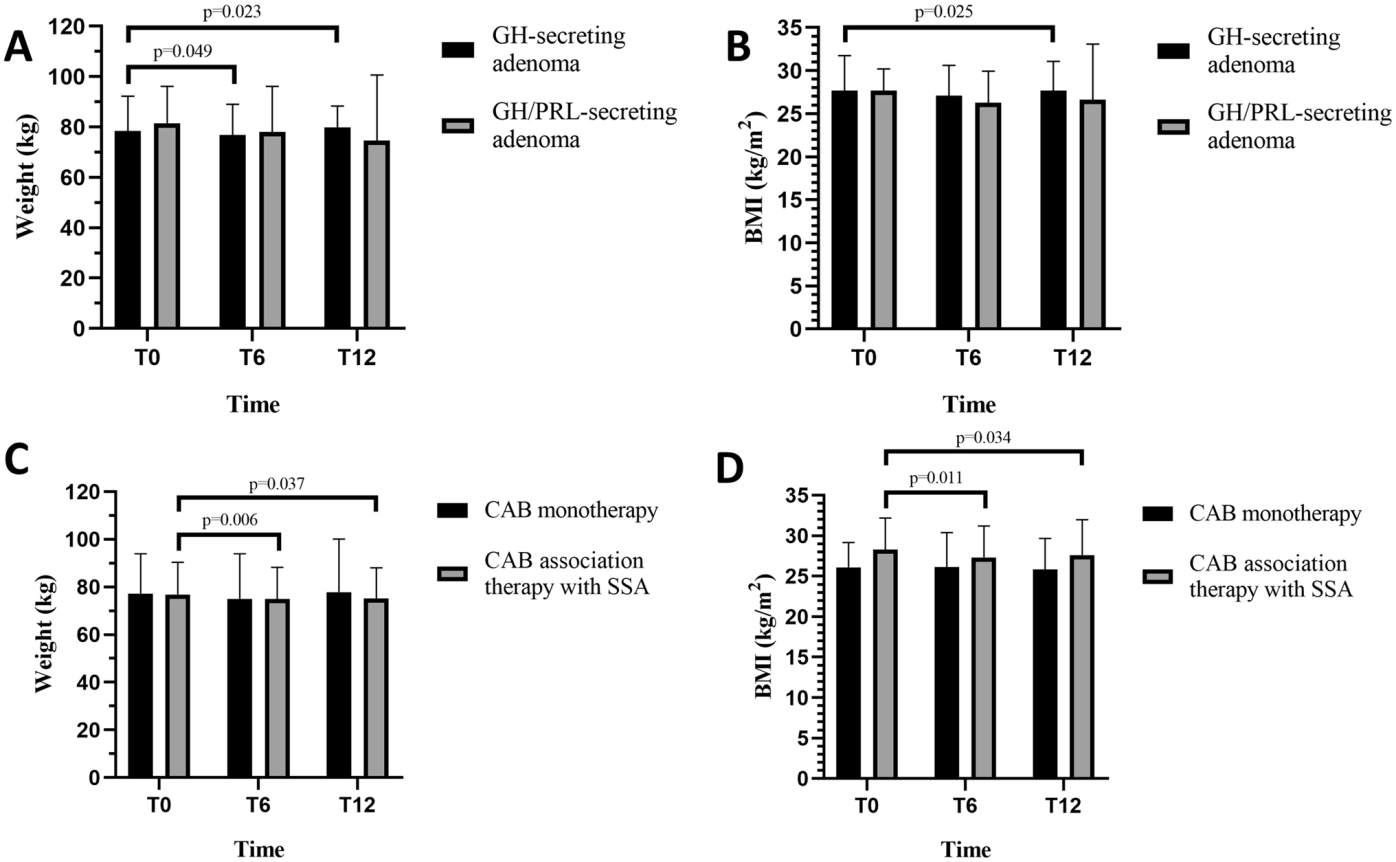
Weight and BMI changes in patients stratified by hormonal secretion type (A–B), and monotherapy or combination therapy (C—D). The number of patients evaluated at each time point is shown in Table 1. *GH* Growth hormone, *PRL* Prolactin, *CAB* Cabergoline, *SSA* Somatostatin analog

At T12 both weight and BMI were significantly lower compared to T0, although not significantly different compared to T6 and this finding was confirmed even taking into account histological examination, ongoing medical therapy and glycemic status (Fig. 2, Table 2). Finally, patients with suppressed PRL values at T12 showed a significant reduction in weight (p = 0.033) and BMI (0.037), as well as in FBG compared to T0 (T0 vs T12, 93 [86–105] vs 89 [78–102] mg/dL, p = 0.049), although no further change was noted from T6 to T12.

## Discussion

To the best of our knowledge, this is the first study to investigate the metabolic effects of CAB in the acromegaly population. Specifically, our results confirm that CAB is a good therapeutic option for achieving good disease control but with modest metabolic impact.

In our study, following the initiation of CAB, a significant decrease in median IGF-I values was observed at 6 and 12 months compared to baseline, despite no significant differences in median GH levels. The discrepancy observed between IGF-I and GH is not surprising: indeed, IGF-I better reflects the daily overall exposure to GH and therefore is a better predictor of the overall disease activity [18].

This finding is also in line with what previously reported in patients undergone neurosurgery [19] and in patients treated with SSAs [20], and it could be attributable to biological factors such as sex, glucose metabolism, and GH receptor polymorphisms as well as the consequence of using different measurement techniques [21, 22].

The incidence of glycemic alterations in patients with acromegaly is higher compared to that of the general population, with over 50% of patients developing either DM or IGM [23].

In choosing the appropriate treatment for patients, it is thus important to consider the effects that therapy may have on glycemic control and weight, as these are significant determinants of the quality and life expectancy of acromegaly individuals [24, 25].

In this regard, DAs have shown to significantly reduce FBG levels, regardless of the presence of hyperPRL.

In several studies, a favorable effect of CAB on the glycemic profile of patients with prolactinoma has also been observed, particularly an improvement in surrogate indices of insulin resistance, insulin sensitivity, and pancreatic β-cell function after only 6 months of therapy, regardless of PRL values [10, 12, 14, 26].

In our study, the metabolic impact of CAB was less evident than hypothesized and considering the entire cohort, no significant variations were observed in terms of FBG levels and HbA1c at 6 or 12 months.

Compared to subjects with prolactinomas, it can be speculated that in acromegaly patients, the action of CAB may require a longer treatment period in order to appreciate its positive impact on glucose metabolism. Indeed, it is well known that GH is a key hormone in glycemic counter-regulation and the primary culprit for alterations in glycemic homeostasis in these patients [27]. CAB in monotherapy, in acromegaly, is significantly less effective in normalizing GH/IGF-I levels, and this certainly affects its metabolic action. Only 40% of patients are actually able to normalize GH secretion; in 60% of cases, patients still have pathological levels of GH, negatively impacting the glycemic profile [28].

In our study, a notable difference in FBG levels at T12 compared to T0 was observed in non-diabetic patients, contrasting with previous findings in subjects with prolactinomas. In the latter group, the impact on glycemic control was shown to be more prominent among patients with IGM or DM, occasionally leading to the normalization of glycemic tolerance or a dosage reduction in antidiabetic therapy [12].

Then, in acromegaly patients, it is possible that CAB might modulate glycemic parameters more effectively in subjects who likely retain a better-preserved β-cell pancreatic function.

Although CAB has shown suboptimal performance in improving the glycemic profile, our results demonstrate a significant weight and BMI reduction at 6 months, sustained at 12 months, more noticeable in the subgroup of overweight patients. However, there were no significant differences in the percentage of weight loss based on the initial BMI class. Therefore, it is likely that most patients, regardless of being obese or overweight, can benefit from CAB therapy. An important aspect to note is that no patient with DM was on therapy with drugs like GLP1-RA or SGLT2-i, antidiabetic medications known for their weight loss effects [29]. This exclusion ensures the elimination of significant pharmacological confounders.

Similarly, patients with untreated concurrent hormonal deficiencies, particularly those with secondary hypogonadism, were excluded from the analysis, thereby also reducing the impact of restoring eugonadism on the evaluation of glycemic and weight variations.

The effect on weight has also been documented in patients with prolactinomas. Specifically, in some studies, a significant weight loss was observed as early as 3–6 months [13, 30], while in others, it took more than 60 months of treatment to detect it [12].

The weight loss observed in prolactinomas correlates with elevated baseline PRL levels and male gender [30], but it is independent of the improvement in other metabolic parameters [12, 26].

In our study, no association was demonstrated between PRL levels at T0 and weight changes. However, it is worth noting that most of our patients had either normal or suppressed PRL values already at baseline.

It is therefore likely that the mechanisms behind the weight loss observed in acromegaly individuals are somewhat different from those in patients with isolated hyperPRL. Furthermore, weight loss does not seem to correlate with the state of active or controlled disease.

In more recent studies, it has been shown that even reduced levels of PRL compared to the normal range can have a negative impact on weight, glycemic profile and lipid levels, promoting an increased risk of metabolic syndrome, similarly to an excess of PRL [31].

In our investigation, this data did not find confirmation; indeed, analyzing subjects with inhibited PRL levels, our results showed significant reductions in FBG levels at T12. Similarly, patients with suppressed PRL levels showed significant weight loss at both T6 and T12. As previously observed at baseline, however, the majority of patients in our cohort exhibited suppressed PRL levels following initiation of CAB therapy, with only three patients showing normal levels. The small sample size could be responsible for the obtained results, but the suppression of PRL values, anyway, did not appear to be detrimental to metabolic status. In any case, it is worth noting that the definition of hypoprolactinemia (hypoPRL) is a relatively new nosological entity [32] and has not yet been fully codified as of today, since different studies have proposed values lower than 7 or 5 µg/L, or even undetectable levels for the cutoff [31, 33, 34]. Therefore, the adverse effects of low PRL levels on cardiovascular and metabolic risk are likely dependent on the specific definition of hypoPRL being used.

Stratifying subjects based on hormone secretion, no significant variations were observed in metabolic parameters or disease hormone compensation. This data confirms that CAB is effective regardless of PRL co-secretion [3]. Moreover, in the subgroup with GH-secreting adenoma, a significant reduction in weight was observed at 6 and confirmed at 12 months. The metabolic effect of CAB, when present, appears to primarily affect anthropometric parameters but does not seem to be progressive, at least in the short-term period.

Our results confirm the efficacy of CAB in hormonal control, administered alone or in combination with SSAs or PEG. SSAs are known to inhibit pancreatic secretion of insulin and glucagon, promoting the development of metabolic alterations in euglycemic patients, and aggravating the already partially impaired pancreatic function in patients with IGM or DM.

In recent studies, the impact on metabolism of SSAs, at least first-generation ones, has been reassessed.

It appears that their effect is actually marginal, as their use is associated with a transient reduction in insulin levels, without, however, significant effects on glycemic homeostasis [35]. On the other hand, pasireotide is a second-generation SSA known to significantly worsen glucose metabolism, with an average increase of about 0.4–0.5% in HbA1c levels [36, 37]; anyway, no patients in our cohort were on treatment with such a drug.

Finally, it is well known that PEG primarily presents favorable effects on glycemic homeostasis, reducing FBG levels even after achieving good disease control [38].

In our study, the type of treatment being administered did not show advantages in terms of either FBG or HbA1c levels, but it is worth noting that only 3 people were on PEG treatment.

In the subgroup receiving combination therapy with SSAs, a positive effect on weight was observed, with a significant weight loss at 6 and 12 months compared to baseline, suggesting an influence of other acromegaly therapies, particularly SSAs, on weight [39].

Although this is the first study to evaluate the metabolic impact of CAB in patients with acromegaly, it has some limitations. Firstly, the sample size was rather small. Moreover, its retrospective nature did not allow the assessment of parameters such as the lipid profile, insulin, HOMA-IR, as well as other anthropometric measures such as waist circumference, hips, waist-to-hip ratio or body composition. Similarly, data on blood pressure, as well as information about potential significant changes in dietary habits were not available for most patients. Finally, there was no control group, and the follow-up was limited to a period of 12 months.

In conclusion, our results confirm the efficacy of CAB in providing a significant improvement in the biochemical disease control but do not demonstrate a marked benefit on glucose metabolism of acromegaly patients. Anyway, in such patients, CAB appears to have a rapid effect on weight and BMI, with significant changes noticeable as early as 6 months and persisting for at least 12 months.

Considering the importance of preventing and managing complications related to the excess of GH and/or IGF-I and considering the established efficacy of CAB in other clinical settings, further prospective studies involving larger cohort and longer observation periods are necessary to evaluate more deeply the potential clinical and metabolic benefits of CAB in acromegaly disease.

## Data Availability

The data sets generated during and/or analyzed during the current study are not publicly available but are available from the corresponding author on reasonable request.
